# Quantitative evaluation of OCT angiography images in healthy and glaucomatous subjects through a novel approach: exploring inter-image variability

**DOI:** 10.1038/s41433-024-03038-9

**Published:** 2024-04-13

**Authors:** Paola Cassottana, Costanza Iester, Laura Bonzano, Carlo E. Traverso, Michele Iester

**Affiliations:** 1 Clinica Oculistica, IRCCS Ospedale Policlinico San Martino, Genoa, Italy; 2 Department of Neurosciences, Rehabilitation, Ophthalmology, Genetics, Maternal and Child Health (DiNOGMI), University of Genoa, Genoa, Italy

**Keywords:** Optic nerve diseases, Prognostic markers

## Abstract

**Purpose:**

This study aims to investigate inter-image intra-observer variability of macular, and optic disc (ONH) microvasculature measurements of glaucomatous and normal subjects using Swept-Source Optical Coherence Tomography Angiography (OCT-A) (OCT Topcon ImageNet 6; DRI OCT Triton, Topcon Corporation, JAPAN) - based imaging data analysis and processing with a newly made quantitative approach.

**Methods:**

A total of 20 glaucomatous and 20 healthy eyes underwent three OCT-A scanning of the ONH and macula. Macular and papillary and peripapillary vascular networks were calculated. For each eye, eighteen scans were analyzed using a novel approach: custom MATLAB 2021b scripts were employed for imaging analyses. Grayscale distribution was performed using the histcounts MATLAB function with 51 bins. For all layers, the vascular layer coefficient of variation (vl CoV) of the three measures were performed. The vl CoV difference between the two groups was analyzed by Student t-test.

**Results:**

In glaucomatous eyes, the vl CoV ranged from 4.49% to 8.54%, while in the control group from 3.58% to 8.32%. Both groups exhibited higher CoVs when assessing the optic disc. The papillary and macular microvasculature reproducibility was comparable between groups.

**Conclusions:**

Utilizing Swept-Source OCT-A images our study has identified an easy and reproducible method that appears to be fast and can assist physicians in assessing macular and ONH perfusion with less inter-image variability, particularly in the 70 μm superficial area of the optic disc. The high reliability obtained suggested that this method could be useful as early clinical biomarker.

## Introduction

Glaucoma, a leading cause of irreversible blindness worldwide, is often diagnosed at advanced stages when significant and irreversible visual field loss has already occurred. The exact pathogenetic mechanism of damage to retinal ganglion cells (RGCs) and their axons is not fully characterized [1, 2]. While elevated intraocular pressure (IOP) is strongly associated with glaucomatous optic neuropathy, studies have indicated that vascular components may also play a role in the development and progression of glaucoma [3–5]. Ocular perfusion defects have long been implicated in glaucoma’s pathophysiology, although the details remain poorly understood. Low ocular perfusion pressure (OPP), which is expressed as the difference between mean arterial pressure and IOP, has been considered to lead to glaucomatous optic neuropathy via ischemic damage to the optic nerve [6]. Studies that attempt to examine blood flow at the optic nerve more directly have shown alterations in the normal blood supply in glaucoma. The normal blood supply to the optic nerve head mainly derives from two branches of the ophthalmic artery: the superficial layers of the nerve are supplied by arterioles of the central retinal artery (CRA), while the deeper layers are supplied by branches of the posterior ciliary artery (PCA) via the circle of Zinn-Haller.

En-face optical coherence tomography angiography (OCT-A) has emerged as a promising technology for ocular perfusion study. OCT-A provides reproducible and non-invasive imaging of the microvasculature in the macula, optic nerve, and peripapillary retina. Unlike traditional fluorescein angiography (FA), OCT-A does not require contrast agents, as it uses an algorithm to detect motion of blood cells and generate motion-contrast images [7, 8].

Given the impact of glaucoma on quality of life, there is a need to identify early optic nerve head structural changes that could predict visual field defects. Ocular microvascular abnormalities observed in glaucomatous eyes but not in healthy eyes may serve as early biomarkers in pre-perimetric glaucoma, enabling timely diagnosis to prevent functional damage to the visual field.

However, existing technologies face challenges of reproducibility and inter-test variability, which hinder our ability to visualize and quantify blood flow in the optic nerve [9, 10]. Therefore, this study aims to investigate the reproducibility and inter-image variability of macular and papillary microvasculature measurements in healthy and glaucomatous subjects using an OCT-A-based imaging data analysis and processing program.

## Method

### Participants

A total of forty eyes were recruited from the Clinica Oculistica, Policlinico San Martino Hospital IRCCS, University of Genoa, between January 2022 and November 2022. The study adhered to the principles of the Declaration of Helsinki, and written informed consent was given by all participants before performing eye examination as common practice in our hospital. This study is part of the GAUGER project (Prot. GAUGER, CET Liguria: 582/2023). The cohort included twenty glaucomatous eyes from 13 patients (Glaucoma Group, GG) and twenty healthy eyes from 11 subjects (Control Group, CG).

We did not consider enrolling both eyes of the same subject as a limitation since the main focus of our study was to identify the layers where MATLAB 2021b software demonstrated higher reliability in analyzing blood flow in various layers of the retina and choroid.

For all subjects, common inclusion criteria were age ≥18 years old, an open angle on gonioscopy, and best corrected visual acuity better than 20/40.

For the control group, inclusion criteria required participants to have healthy eyes without any ophthalmological or systemic diseases with ophthalmological involvement, while exclusion criteria involved any previous ophthalmic surgeries, including refractive surgery.

The glaucoma group consisted of a mixed population of open-angle glaucoma, including primary open-angle glaucoma (POAG), exfoliative glaucoma, or pigmentary glaucoma. Inclusion criteria for the glaucoma group included a history of untreated IOP ≥ 22 mmHg, open angle on gonioscopy, glaucomatous optic nerve defect confirmed through dilated fundus examination, and evidence of glaucomatous visual field damage based on Hodapp-Parrish-Anderson criteria [11] (MD better than −12.0 dB, ranging from early to moderate defect). All subjects had achieved a target IOP, either through medical, laser therapy, or surgery.

History of previous glaucoma surgery, or uncomplicated phacoemulsification did not represent an exclusion criterion (Table 1). Systemic medications that could potentially impact OPP were considered as exclusion criteria in both study groups.

**Table 1:** Demographics and clinical characteristics of healthy and glaucomatous eyes.

Variables	Normal *n* = *20*	POAG *n* = *12*	Exfoliative Glaucoma *n* = *6*	Pigmentary Glaucoma *n* = *2*
Age, years	49.5 ± 12	68 ± 9	50.5 ± 8	47
Gender. male/female	16/4	2/10	2/4	2/0
IOP, mmHg	15.2 ± 1.8	14.6 ± 3.5	16.8 ± 2.3	16.5 ± 0.5
Vertical cup to disc ratio	0.3 ± 0.1	0.8 ± 0.1	0.7 ± 0.1	0.7 ± 0.1
CCT, μm	528 ± 22	525 ± 20	536 ± 18	552 ± 7
Visual Acuity, decimal	0.9 ± 0.6	0.8 ± 0.3	0.7 ± 1.2	0.9 ± 0.8
Visual Field MD, dB	**–**	−5.36 ± 3.04	−6.13 ± 2.87	−3.58 ± 1.04
Visual Field PSD, dB	**–**	6.35 ± 3.17	7.69 ± 3.01	3.56 ± 1.08

*POAG* primary open angle glaucoma, *IOP* intraocular pressure, *CCT* central corneal thickness, *MD* mean deviation, *PSD* pattern standard deviation.

### Data acquisition

All participants underwent a comprehensive ophthalmological examination, including best-corrected visual acuity, slit-lamp biomicroscopy, gonioscopy funduscopy, and IOP measurements using Goldmann applanation tonometry.

Subsequently, Swept-Source Optical Coherence Tomography Angiography (OCT-A) (OCT Topcon ImageNet 6; DRI OCT Triton; Topcon Corporation; JAPAN) imaging was performed on each eye using the Angio 4.5 × 4.5-mm protocol to cover the macula and optic disc. To investigate the repeatability of the measurements, three sets of macula and optic disc scans were obtained for each eye, at different times, repositioning the head of the participant every time in the same day, resulting in a total of 24 scans per eye. Macular vessel densities were measured in the following areas:

- Superficial region: from the inner limiting membrane (ILM) to inner plexiform layer (IPL), supplied by Central Retinal Artery (CRA);
- Deep region: from IPL to 70 μm below the inner nuclear layer (INL), supplied by CRA;
- Choriocapillaris: from BM to 10 μm below BM, supplied by Posterior Ciliary Artery (PCA).
  Optic disc vasculature was measured in the following layers:
- Vitreous: from ILM to 49,4 μm below ILM, supplied by CRA;
- Radial peripapillary capillaries (RPC): from ILM to 70,2 μm below ILM, supplied by CRA;
- Choroid/disc: from 130 μm below ILM to 390μm below Bruch membrane, supplied by PCA.

### Data analysis

All the imaging analyses were performed in MATLAB 2021b with ad hoc scripts. The OCT acquisitions were renamed automatically to automize analysis. A MATLAB program showed one image at a time, and the expert selected only images with good quality. The assessment of good image quality has been determined by considering the Signal Strength Index (SSI), Signal-to-Noise Ratio (SNR), and Artifact Index, following the manufacturer’s specified values for OCT. Images that were discarded will no longer be considered for the study. The selected images were loaded, and then converted in greyscale intensities (greyscale goes from zero to 255, where zero corresponds to black and 255 to white) because the distribution of greyscale in the picture suggests the vascularity (zero corresponds to no flow and 255 to maximum flow). The gray distribution was performed utilizing the *histcounts* MATLAB function. This function returned the number of pixels in successive intervals called bins. To compare different images, it was necessary to set the same number of bins, moreover, the same intervals were necessary. Intervals were found by performing a preliminary analysis without constraints and then selecting the more appealing bin subdivision. The 0–255 range was divided into bins with the same dimension. The higher the number of pixels, the higher the accuracy, but the lower the continuity between successive bins. Thus, the trade-off between accuracy and continuity returned 51 bins as the best solution. In the subsequent analyses, the first and the last bins will be rejected.

To test reproducibility, the values of the three images from the same subject and layer were compared for each bin. The mean, standard deviation, and coefficient of variation (CoV) of bins among the three images were calculated. For each vascular layer, the subject CoV corresponded to the mean of CoV obtained from bins. Finally, the vascular layer CoV (vl CoV) was measured as the mean of the subject’s CoV. The same procedure was adopted both for healthy and glaucomatous eyes (Tables 2 and 3). To estimate the inter-image variability, the number of pixels with CoV <10% was calculated in all 720 scans (Table 4).

**Table 2:** Mean inter-image Coefficients of Variation (CoV) in Swept-Source (Topcon, DRI OCT Triton) OCT-A assisted macula assessment (%).

Macula scan			
	Control group	Glaucoma group	*p*-value
Superficial plexus	5.52	5.62	0.893
Deep plexus	7.20	7.91	0.359
Choriocapillaris	8.32	8.09	0.800

**Table 3:** Mean inter-image Coefficients of Variation (CoV) in Swept-Source (Topcon, DRI OCT Triton) OCT-A assisted optic nerve head (ONH) assessment (%).

Optic nerve head scan			
	Control group	Glaucoma group	*p*-value
Vitreous	4.21	5.74	0.024
RPC	3.58	4.49	0.071
Choroid/disc	6.67	8.54	0.069

**Table 4:** Mean of number of pixels with CoV <10% for each image.

Macula scan		
	Control group	Glaucoma group
Superficial plexus	81.25%	84.16%
Deep plexus	77.93%	73.96%
Choriocapillaris	72.35%	75.15%
**Optic nerve head scan**		
Vitreous	91.35%	83.71%
RPC	93.65%	90.06%
Choroid/disc	82.5%	70.04%

The difference in CoV between the two groups was analyzed by unpaired Student’s *t* test.

### The novel approach

In this study, we introduce a novel approach for analyzing Swept-Source OCT-A images, aiming to offer a distinctive and efficient method for assessing macular and optic nerve head (ONH) perfusion. Our analysis program, developed in MATLAB 2021b with ad hoc scripts, exploits the premise that black pixels indicate no flow, white pixels represent high flow, and gray pixels represent varying degrees of blood cell movement within vessels. Unlike traditional methods, our approach focuses solely on the intermediate flow levels, providing a more nuanced understanding of blood flow dynamics. By categorizing pixels based on their values and generating a curve to summarize the data, ensuring a more accurate interpretation of the data. This innovative methodology contributes to overcoming challenges related to reproducibility and inter-test variability, enhancing the reliability of our microvasculature measurements. The 51-bin subdivision, determined through a preliminary analysis without constraints, strikes a balance between accuracy and continuity, optimizing the analysis of blood flow in various layers of the retina and choroid.

## Results

**Fig. 1:**
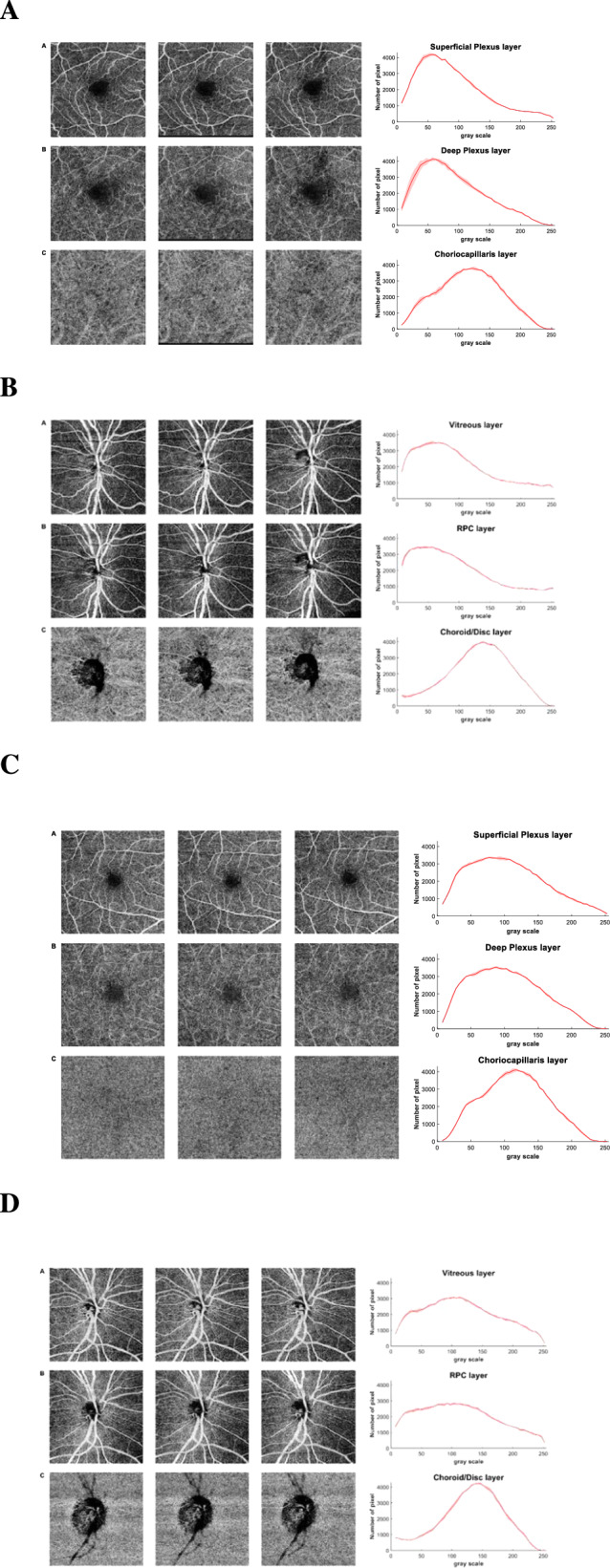
Three Swept-Source (Topcon, DRI OCT Triton) OCT-A scan images. **A** for a representative glaucoma patient in the macular assessment: A Superficial Plexus, B Deep Plexus and C Choriocapillaris layers. On the right, the function distribution with mean and standard deviation of the three scans for each bin. **B** for a representative glaucoma patient in the assessment of the optic disc: A Vitreous, B RPC and C Choroid/disc layers. On the right, the function distribution with mean and standard deviation of the three scans for each bin. **C** for a representative control subject in the macular assessment: A Superficial Plexus, B Deep Plexus and C Choriocapillaris layers. On the right, the function distribution with mean and standard deviation of the three scans for each bin. **D** for a representative control subject in the assessment of the optic disc: A Vitreous, B RPC and C Choroid/disc layers. On the right, the function distribution with mean and standard deviation of the three scans for each bin.

Figure 1a, b show the trends of mean and standard deviation for each layer of a representative subject from the glaucomatous group, and Fig. 1c, d from a subject from the control group. In glaucomatous eyes, vl CoV ranged from 4.49% to 8.54%, while in the control group it ranged from 3.58% to 8.32%. The macular layers obtained the best vl CoV both for glaucomatous and healthy eyes in the superficial plexus of 5.62% and 5.52%, respectively. While, the optic disc layers obtained the best vl CoV in the RPC for both glaucomatous and healthy eyes, 4.49% and 3.58%, respectively. When examining the analysis for each level of gray, CoV <10% was observed in 90.06% of glaucomatous eyes for RPC, 83.71% for the vitreous, 70.04% for the choroid/disc. Similar results were found in healthy eyes, with the best CoV observed in RPC (93.65%), followed by vitreous (91.35%) and choroid/disc (82.5%). In terms of macular analysis, the superficial plexus exhibited the best reproducibility for both glaucoma (84.16%) and healthy (81.25%) groups, followed by the deep plexus with 73.96% for glaucoma and 77.93% for healthy. The choriocapillaris layer showed a reproducibility of 75.15% for glaucoma and 72.35% for healthy individuals.

Regarding ONH blood flow analysis, the RPC and vitreous layers showed the best reproducibility, followed by the choroid/disc and the nerve head layers for both glaucoma and control groups. In comparing the COV of glaucomatous eyes with healthy ones in macular assessment, less inter-image variability was observed in the superficial plexus, followed by choriocapillaris, deep plexus, and outer retina layers. However, no statistically significant difference was found for all the coefficients of variation for each layer (*p* > 0.05), except for the vitreous layer (*p*-value 0.024).

No diagnostic capacity for this novel method was considered in the study.

## Discussion

In recent years, analysis of choroidal and retinal vessels has become easier with the introduction of the OCT-A, an improvement over OCT. OCT-A is a non-invasive imaging technique that relies on the movement of blood cells in the vessels, and it has become a relevant diagnostic tool in clinical practice. OCT-A allows visualization of vessel structure and flow. However, currently, there is no standardized method for analyzing the images, and physicians primarily interpret them qualitatively rather than quantitatively. In a medical retina office, the ability to identify new vessels beneath the retina is crucial in deciding whether to initiate intravitreous treatment. In other pathologies, assessing the flow may be more useful in effectively monitoring patients during follow-up.

Another challenge is that different OCT-A devices utilize different algorithms to generate vascular chorio-retinal maps based on the OCT signal they receive. Consequently, different OCT-A devices may produce varying values for vessel density. Various methods have been described in the literature to address this issue.

Lupidi et al., with the aim of quantitatively analyzing blood flow, developed an automated quantitative technique for visualizing and analyzing macular vascular perfusion using optical coherence tomography angiography (OCT-A). They analyzed and compared the superficial capillary plexus with the deep capillary plexus, and significant differences were found for the perimetry, surface and major axis of the foveal avascular zone. They improved the software, known as AngioQuant™, facilitating a highly reliable and reproducible quantitative assessment of various findings that were previously limited to qualitative analysis [12].

Hosari et al. conducted a study to assess the reliability of macular microvasculature measurements using Heidelberg Spectralis II optical coherence tomography angiography (OCT-A) in conjunction with the semiautomated vessel density software EA-Tool. They evaluated the vessel density by dividing the OCT image in 12 segments for the superficial vascular plexus, intermediate capillary plexus and deep capillary plexus. Thanks to this combination, they found good or even excellent ICCs in 75% of all analyzed segments of the vascular layers [13].

Our proposed method analyses Swept-Source (Topcon, DRI OCT Triton) OCT-A images by exploiting the premise that black pixels indicate no flow, white pixels represent high flow, and gray pixels represent varying degrees of blood cell movement within vessels.

For each image, each pixel was assigned to a subgroup based on its value. Subsequently, a curve was generated to summarize all the data obtained from each subgroup. The analysis focuses only on the gray pixels, excluding the black pixels representing no flow, and the white pixels indicating high flow, thereby considering only the intermediate flow levels.

When analyzing the microvasculature in the ONH, less inter-image variability was observed in the 70 μm superficial area of the optic disc, which includes the RPC and vitreous layers for the analysis of the optic disc, and in the superficial and choriocapillaris layers for macular blood flow assessment in both glaucomatous and healthy eyes. These layers may be the focus for the analysis of blood flow defects and could serve as promising parameters for diagnosing glaucomatous damage and early biomarkers in glaucoma pathogenesis. The selective vascularization at these layers could be regarded as a potential biomarker to identify eyes at risk of developing glaucomatous optic neuropathy before functional alterations become apparent in perimetric examinations. *However, currently, there is no real clinical parameter available. This study aims to confirm the feasibility of using the distribution of gray levels to derive a clinical metric. The next step involves utilizing reliable data to establish such a metric. Moreover, this clinical parameter may serve as a biomarker for depicting ocular diseases*.

There are some limitations to the current study. Firstly, the quality of the images is affected by the collaboration of the subjects, which is crucial for this type of analysis. Saccades in the image pose a problem for the analysis since the pixels are interpreted as chorio-retina flow rather than eye movements. Therefore, any lines resulting from saccades should be excluded from the analysis to ensure an accurate interpretation of the data.

The small number of eyes enrolled in the control and glaucoma groups may be considered a limitation. However, a large cohort is not always necessary in reproducibility studies. The focus of reproducibility studies is typically on assessing the consistency and reliability of the measurements rather than generalizability to a larger population. Nonetheless, it is valuable to acknowledge the sample size as a potential limitation when interpreting the study’s findings.

Furthermore, this study did not account for potential confounding factors such as age, systemic comorbidities, or medication use, which could influence ocular blood flow and introduce bias.

By addressing these limitations, future studies can build upon the current findings and provide further insights into the assessment of OCT-A in glaucoma research.

The current findings argue for a pathogenetic role of ocular blood flow [3] and confirm that OCT-A could be a useful technique to detect early glaucoma [14].

Further studies could explore the reproducibility of the method by acquiring images on different days. In our study, we focused on investigating the intra-subject variability, but in the future, it would be valuable to study inter-subject variability as well. Additionally, by increasing the sample size, a comparison between healthy subjects and glaucoma patients could be conducted to assess potential differences.

The developed analysis program could also be utilized to quantify flow within the retinochoroidal microvasculature based on the percentage of white pixels, providing insights into potential quantitative differences in vascularization between glaucomatous and healthy eyes. Additionally, investigating the correlation between blood flow measurements and global indices from visual field tests could help understand the relationship between vascular integrity of the optic nerve and functional progression in glaucomatous patients, shedding light on whether vascular alterations are causes or effects of optic nerve damage in glaucoma.

In conclusion, utilizing Swept-Source (Topcon, DRI OCT Triton) OCT-A images our study has identified an easy and reproducible method that appears to be fast and can assist physicians in assessing macular and ONH perfusion.

## Summary

### What was known before

- The loss of capillary around the ganglion cell could be related to a primary reduction of the vessels or to a secondary loss of ganglion tissue, however in glaucoma there is a reduction of capillary tissue.

### What this study adds

- Blood perfusion can be easily assessed by using angio-OCT images. this new approach could help the physician to better assess the stage of the disease.

## Data Availability

The data that support the findings of this study are not openly available due to reasons of sensitivity and are available from the corresponding author upon reasonable request.
